# Preparation of PolyHIPE Scaffolds for 3D Cell Culture and the Application in Cytotoxicity Evaluation of Cigarette Smoke

**DOI:** 10.3390/polym11060959

**Published:** 2019-06-02

**Authors:** Peijian Sun, Song Yang, Xuehui Sun, Yipeng Wang, Yunzhen Jia, Pingping Shang, Haiying Tian, Guozheng Li, Ruyang Li, Xiaobing Zhang, Cong Nie

**Affiliations:** 1 Key Laboratory of Tobacco Chemistry, Zhengzhou Tobacco Research Institute of CNTC, No.2 Fengyang Street, Zhengzhou 450001, China; pjsunztri@163.com (P.S.); xuehui_sun1234@aliyun.com (X.S.); yipeng_w@sina.com (Y.W.); xiaojia2003@163.com (Y.J.); shangpingpingan@126.com (P.S.); liruyang1992@163.com (R.L.); zhangxiaobing985@sohu.com (X.Z.); 2 Technology Center, China Tobacco Henan Industrial Co., Ltd., Zhengzhou 450000, China; haiyingflying@163.com (H.T.); guozheng_li@126.com (G.L.)

**Keywords:** polyHIPE, 3D cell culture, cytotoxicity evaluation, cigarette smoke, polystyrene

## Abstract

Polystyrene-based polyHIPE (polymerized high internal phase emulsion) materials were prepared by the copolymerization of styrene and divinylbenzene in the continuous phase of a HIPE. The resultant polyHIPE materials were found to have an open-cellular morphology and high porosity, and the polyHIPE structure could be well adjusted by varying the water/oil (W/O) ratio and the amount of emulsifier in the HIPE. Cell culture results showed that the resultant polyHIPE materials, which exhibited larger voids and connected windows as well as high porosity, could promote cell proliferation on the 3D scaffold. A 3D cell cytotoxicity evaluation system was constructed with the polystyrene-based polyHIPE materials as scaffolds and the cigarette smoke cytotoxicity was evaluated. Results showed that the smoke cytotoxicity against A549 cells is much lower in the 3D cell platform compared to the traditional 2D system, showing the great potential of the polyHIPE scaffolds for 3D cell culture and the cytotoxic evaluation of cigarette smoke.

## 1. Introduction

Cigarette smoke, which is an extremely complex aerosol stream consisting of more than 5000 identified chemicals, is well-recognized as one of the most important causes of lung cancer, chronic obstructive pulmonary disease and cardiovascular disease [1,2]. The issue of smoking on population health has become a growing concern for the public and the government. A variety of in vitro cytotoxicity assays, such as the neutral red uptake (NRU) assay, 3-(4,5-dimethyl-2-thiazolyl)-2,5-diphenyl-2H-tetrazolium bromide (MTT) assay, cell counting kit-8 (CCK-8) assay, lactate dehydrogenase (LDH) assay, as well as Ames Salmonella reverse mutagenicity assay and in vitro micronucleus test have been applied to evaluate the potential health risks and harmful effects of cigarette smoke [3,4,5,6,7]. The in vitro study of smoke-induced cellular effects would not only benefit the understanding of smoke toxicity but also lead to a better understanding of smoking-induced disease mechanisms. The total particulate matter (TPM) of smoke, cigarette smoke condensate (CSC), gas vapor phase (GVP) and/or the whole smoke (WS) were exposed to the cells in vitro in order to study the smoking-induced cellular effects [3,4,5,6,7]. For example, the in vitro cytotoxicity and genotoxicity of TPM with reduced toxicant yields were evaluated by the NRU assay, Ames Salmonella reverse mutagenicity assay and in vitro micronucleus test [6]. Results showed that the tobacco substitute sheet could reduce TPM cytotoxicity in the NRU assay, and the blend-treated tobacco provided a reduced bacterial mutagenicity in the Ames Salmonella reverse mutagenicity assay.

Conventionally, the cytotoxicity evaluation of cigarette smoke is mainly based on two-dimensional (2D) cell culture systems, in which cells are monolayer cultured directly on a tissue culture plate. For example, Li [7] compared the toxicological effects of the in vitro whole smoke (WS) and TPM exposure on Chinese hamster ovary cells. Their results indicated that the WS exposure cytotoxicity is greater than that of TPM exposure. The conventional 2D cell culture base cytotoxicity evaluations of cigarette smoke have benefited a greater understanding of smoke toxicity and the mechanisms of smoking-induced disease. However, the environments in conventional 2D cell culture systems have a quite large difference from the natural surroundings of the cells in the living tissues, in which cells are organized in three-dimensional (3D) structure with cell-extracellular matrix (ECM) interactions, cell-cell interactions, cell migration and invasion, as well as the transfer of nutrients and wastes [8,9]. Due to the loss of these special characteristics, 2D cell culture systems were oversimplified and could poorly simulate the complex tissue architectures, thus the cytotoxicity evaluation results based on 2D cell culture systems is accompanied by great limitations.

In vitro 3D tissue models have also been utilized as a feasible platform to evaluate the cytotoxicity of smoke. For example, a respiratory epithelial model of the commercially available EpiAirway, which is constructed by primary human tracheal-bronchial epithelial cells, was exposed to e-cigarette aerosol and/or cigarette smoke in order to compare the biological effects of e-cigarettes and conventional cigarettes [10]. Results showed that the tissue viability and barrier function decreased significantly and that the presence of DNA damage marker γ-H2AX increased greatly upon the exposure to cigarette smoke while a negligible change of tissue viability was observed during exposure of the tissue to e-cigarette aerosol [10]. Compared to 2D cell culture systems, the 3D tissue model could better mimic the in vivo exposure route of cigarette smoke. However, the high cost and complex operation of the 3D tissue models have limited the wide application of these in vitro 3D tissue models. Alternatively, a 3D cell culture approach also exhibits features which are much closer to the complex in vivo environment and have proven to be more realistic for translating the study findings for in vivo application. The 3D culture approach has taken us a step closer to the in vivo conditions due to its advantage in decreasing in the gap between cell cultures system and the cellular physiology. The artificial 3D platforms could provide an additional dimension for cellular proliferation and interaction, which would greatly benefit the cell morphology, the cell spatial organization, as well as cell-cell and cell-ECM interactions [11,12,13], better mimicking the characteristics of ECM to reflect in vivo conditions. For instance, the mutual integration of signaling pathways, high level of interleukin (IL)-6 and IL-8 secretion, and enhanced ECM deposition for better biomarker expression were observed when cells are allowed to grow in 3D as compared with their monolayer counterparts [14,15,16]. Cells in a 3D environment are good models as “near-to-in vivo” systems and give us useful insights from a variety of aspects [17]. They can serve as a cost-effective platform to provide a better and more realistic predictive value for safety and risk assessment. Therefore, 3D cell culture platforms that could better recapitulate the in vivo tissue functions that have been developed. Many biocompatible 3D scaffolds have been developed as 3D cell culture systems, especially for the application in tissue engineering. These scaffolds are structured to mimic the characteristics of ECM to reflect in vivo conditions, helping the cells to maintain a more representative structure, gene expression and functions. Cells could grow layers on the surface and inside the porous scaffolds. One of the extremely relevant approaches is hydrogel system that created from the natural or synthetic polymers [18,19,20]. Cells are encapsulated in soft gels to grow and form more physiologically relevant 3D constructs. 3D printed scaffolds [21,22] and porous polymeric microspheres based on the biodegradable polymers [23,24], such as poly(lactic acid) (PLA), poly(lactic-co-glycolic acid) (PLGA) and polycaprolactone, could also serve as a 3D cell culture platform. 3D structure meshes based on electrospun fiber represent another typical platform for 3D cell culture, in which cells grow in and around the fibrous 3D topography [25].

Amongst the varied scaffolds applied for 3D cell culture, porous polymers derived from high internal phase emulsions (polyHIPE) have been widely investigated due to their high porosity and controllable morphology that benefit the cell growth [26,27,28,29,30,31,32,33,34,35]. For example, biodegradable porous scaffolds, such as PLGA [33], polycaprolactone (PCL) [34], gelatin [35], were prepared by the high internal phase emulsion templating method and demonstrated great potential for tissue engineering. Furthermore, polystyrene-based polyHIPE materials [36,37], which have a similar chemical composition to conventional tissue culture plate and appropriate mechanical properties for routine handling, were also demonstrated to be suitable for 3D cell culture applications. For the fabrication of polystyrene-based polyHIPE, a water-in-oil (W/O) high internal phase emulsion (HIPE) was prepared, followed by the polymerization of the external phase which contains polymerizable monomers, such as styrene (St) and divinylbenzene (DVB). The internal water phase acts as the porogen and is removed after polymerization of the HIPE, resulting in spherical macropores (named “voids”, typically up to 10 µm in size). The manipulation of the initial emulsion would help to control the void size for excellent scaffold reproducibility and cell growth. Each void is interconnected with small holes (named “windows”, usually 1–2 µm in size), which are resulted from the contracting and rupturing of the thin external phase film during polymerization [38]. The porous PolyHIPE materials have high porosity and a large number of spherical voids that are highly connected with small window throats, which would facilitate nutrient and waste transfer for cells growing in the materials. These 3D platforms have been demonstrated as useful for 3D cell growing, with the great application prospects in the field of tissue engineering, regenerative medicine and drug discovery. However, the application of such 3D cell culture platforms in the field of cytotoxicity evaluation of cigarette smoke is rarely reported.

In this paper, we aimed to construct an easily fabricated 3D cell cytotoxicity evaluation system based on the porous scaffolds. Due to their high porosity, open-cell structure with connected windows, and feasible fabrication, polystyrene-based polyHIPE materials were chosen as scaffolds. The polystyrene-based polyHIPE materials were prepared by the copolymerization of St and DVB in the continuous phase of a HIPE. The influence of the W/O ratio and the emulsifier amount on the structure of the resultant polyHIPE materials were investigated. Then, the 3D cell culture and cell proliferation of carcinomic human alveolar basal epithelial cells (A549) were performed in order to demonstrate the potential of the polyHIPE as scaffolds for 3D cell culture. Finally, with the polystyrene-based polyHIPE materials as scaffolds, a 3D cell cytotoxicity evaluation system was constructed and the cigarette smoke cytotoxicity was evaluated and compared with the conventional 2D platform.

## 2. Materials and Methods

### 2.1. Materials

St and DVB were supplied by Sigma-Aldrich (Shanghai, China) and passed through basic alumina columns before being used. The 2,2′-azobis(2-methylpropionitrile) (AIBN), sorbitan monooleate (Span 80) was obtained from Shanghai Aladdin Bio-Chem Technology Co., LTD (Shanghai, China) and used as received. The 3R4F reference cigarettes were purchased from the Tobacco and Health Research Institute of the University of Kentucky (Lexington, KY, USA). The Cell counting kit-8 (CCK-8) and lactate dehydrogenase (LDH) cytotoxicity assay kit were supplied by Dojindo Molecular Technologies (Mashikimachi, Japan). The human lung adenocarcinoma cell line A549 was obtained from the Cell Bank of Shanghai Institute of Cell Biology, Chinese Academy of Sciences (Shanghai, China).

### 2.2. Preparation of the PolyHIPE Scaffolds

**Figure 1:**
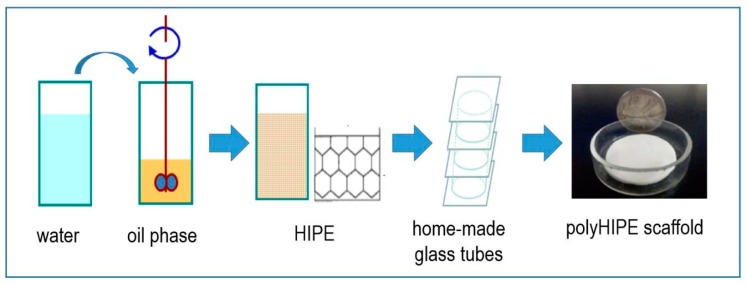
Synthesis pathway of the porous polyHIPE scaffolds.

Porous polystyrene-based polyHIPE scaffolds were prepared using the high internal phase emulsion (HIPE) templating method with Span 80 as emulsifier. Water acted as the porogen and was dispersed in the organic phase which contained polymerizable St and DVB monomers to form a stable HIPE. After polymerization of the continuous phase of the HIPE, the porogen of water was removed and resulted in the highly porous polyHIPE materials. Typically, 0.25 g of AIBN was dissolved in an organic phase consisting of St (5.0 mL), DVB (0.80 mL) and Span 80 (2.90 mL), and then 34.8 mL of water was added dropwisely into the organic phase under stirring at 300 rpm. The mixture was stirred at 500 rpm for another 30 min after the entirely addition of the aqueous solution to form a stable HIPE. The prepared HIPE was subsequently transferred into home-made glass tubes with a 34 mm diameter and polymerization for 24 h at 60 °C (Figure 1). The porous polyHIPE scaffolds were obtained and washed thoroughly by Soxhlet extraction with isopropanol and water as solvents for 24 h before being dried in a vacuum.

Various batches of formulation (as listed in Table 1) were performed and the effect of the W/O ratio and the emulsifier amount on the structure of the resultant polyHIPE materials were investigated.

### 2.3. Characterization of PolyHIPE Scaffolds

The morphology of the polyHIPE scaffold was characterized with a JSM-6700 field-emission scanning electron microscope (SEM) (JEOL, Tokyo, Japan). The samples were mounted directly onto the SEM sample holder using double-sided sticking tape and were then sputter-coated with gold in vacuum prior to measurements. The average void size (D) was determined by statisticitcal calculation from the SEM images [39]. Mercury intrusion porosimetry was performed on a Micrometrics Autopore IV 9500 system (Micromeritics, Norcross, GA, USA) in order to determine the interconnected window diameter (d), total pore volume and porosity. Nitrogen adsorption/desorption measurements were performed on a Micrometrics Tristar II 3020 (Micromeritics, Norcross, GA, USA) gas sorptometer with the sample maintained at −196 °C using liquid nitrogen. Samples were degassed at 80 °C for 10 h under vacuum prior to isotherm determination. The surface area was cumulated by the standard Brunauer-Emmet-Teller (BET) method with the nitrogen adsorption isotherms. Average pore size (<Dw>) was analyzed via the Barrett–Joyner–Halenda (BJH) method by utilizing nitrogen adsorption isotherms.

### 2.4. Cell Culture and Cell Proliferation

Cell culture: The human lung adenocarcinoma cell line A549 was used in this study. The culture medium was RPMI-1640 (Solarbio, Beijing, China) supplemented with 10% fetal bovine serum (Gibco, New York, NY, USA), 100 units/mL of penicillin and 100 µg/mL of streptomycin (Solarbio, Beijing, China). Cells were cultured at 37 °C in a 5% CO_2_ humidified incubator (HERAcell 240, Thermo Scientific, Langenselbold, Germany). The culture medium was refreshed every 2–3 days. When cells had grown to confluence, they were harvested using 0.25% trypsin solution. Prior to cell culture, the polyHIPE scaffolds were sterilized by soaking in 75% ethanol for 12 h, washed with sterile phosphate buffered saline solution (PBS) three times, and exposed to UV light for 6 h.

Cell Proliferation: The polyHIPE scaffolds with a diameter of 34 mm and thickness of 2 mm were placed into a 6-well tissue culture plate (TCP) with 2.0 mL of grown medium and incubated for 12 h. Then, A549 cells suspension were seeded onto the top of the pre-wetted polyHIPE scaffolds at a density of 2.5 × 10^5^ cells per well. At determined time intervals, the polyHIPE scaffolds were transferred into a new pre-cultured 6-well plate and the cell proliferation was evaluated by CCK-8 assay. 2.0 mL of RPMI-1640 medium supplemented with 10% CCK-8 was added to the 6-well plate and incubation for an additional 1.5 h, then 0.20 mL of the culture medium was transferred into a new 96-well plate to measure the optical density (OD) at 450 nm using a Model 680 microplate reader (Bio-Rad Laboratories, Tokyo, Japan). For comparison, the cells were also cultured directly on a 6-well tissue culture plate. Experiments were performed in triplicate for each sample and the average value was used.

### 2.5. Cytotoxicity Evaluation of the Cigarette Smoke

#### 2.5.1. Cigarette Smoke TPM Collection

To produce the cigarette smoke, 20 3R4F reference cigarette were used. The cigarettes were smoked under the standard ISO smoking regime with a puff volume of 35 mL, puff duration of 2 s, and puff interval of 60 s on a M-450 linear smoking machine (Cerulean, Milton Keynes, UK). The TPM in the mainstream cigarette smoke was collected by trapped the smoke on a Cambridge filter pad (Whatman, Buckinghamshire, UK). TPM on the Cambridge filter pad was extracted into dimethylsulfoxide (DMSO) with a TPM concentration of 10 mg/mL. The TPM solutions were then filtered through a sterile cheesecloth and stored frozen at −80 °C.

#### 2.5.2. Cytotoxicity Evaluation of Cigarette Smoke

Cytotoxicity evaluations of cigarette smoke against A549 cells were performed using polyHIPE as scaffolds for 3D cell culture. For comparison, cytotoxicity evaluations of cigarette smoke were also performed with routine 2D cell culture directly on tissue culture plate. Experiments were performed six times for each sample and the average value was used.

Typically, polyHIPE scaffold was placed into a 96-well plate. A549 cells were seeded onto the top of the polyHIPE scaffold at 1.0 × 10^4^ cells per well in 0.10 mL of growth medium. After incubated for 4 h, an additional 0.05 mL of growth medium was added. After culturing for 24 h, cells were treated with 40–280 μg/mL TPM solutions. The negative control cells were treated with 28 μg/mL DMSO solution, which is in accordance to the DMSO amount in the 280 μg/mL TPM expose experiment.

CKK-8 Assay: TPM exposed cells were further incubated for 24 h. Then, the medium was replaced by 0.15 mL RPMI-1640 medium containing 10% CCK-8 and incubation for an additional 2 h, 0.10 mL of the culture medium was taken to measure the optical density at 450 nm using a Model 680 microplate reader (Bio-Rad Laboratories, Tokyo, Japan). The cell viability was normalized to that of A549 cells cultured with 28 μg/mL DMSO.

LDH assay: Cytotoxicity of the TPM was also quantified by the measurement of LDH released in the medium by using an LDH cytotoxic test kit according to manufacturer’s protocol. Briefly, after 24 h incubation with the expose to 40–280 μg/mL TMP, 50 μL of the supernatant from each well was transferred to a new 96-well plate for the LDH assay and mixed with 50 μL of LDH reagent. The samples were incubated at room temperature for 0.5h protected from light. Stop solution was added to each well and the optical density at 490 nm was measured using a Model 680 microplate reader (Bio-Rad Laboratories, Tokyo, Japan).

## 3. Results and Discussion

### 3.1. Preparation of the PolyHIPE Materials

#### 3.1.1. The W/O Ratio

**Figure 2:**
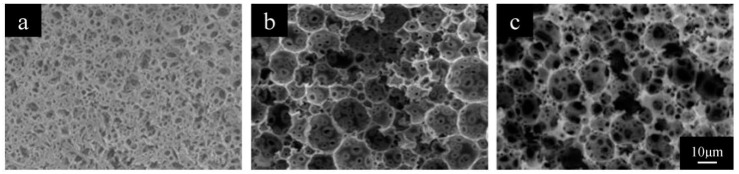
SEM images of PolyHIPE Scaffolds fabricated with W/O ratio of (**a**) 80/20, (**b**) 85/15 and (**c**) 90/10.

The W/O ratio is one of the most important parameters that has a great influence on the HIPE stability, which contributes to the structure of the resultant polyHIPE materials. Figure 2 showed the typical SEM images of the polyHIPE materials prepared with different W/O ratio. All the polyHIPE materials exhibited open-cellular porous structures. Much larger open voids and interconnected “windows” could be observed upon the W/O ratio increasing from 80/20 to 90/10. The average sizes of the voids were 6.5 μm, 11.2 μm and 12.0 μm for the polyHIPE scaffolds prepared with W/O ratio of 80/20, 85/15 and 90/10, respectively, which were listed in Table 2 (Formulations 1–3).

**Figure 3:**
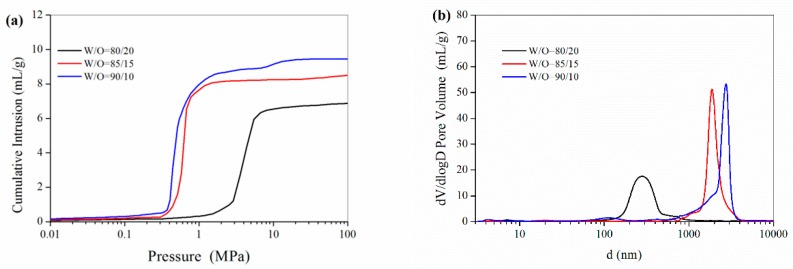
(**a**) Cumulative mercury intrusion traces and (**b**) Size distribution of the interconnected windows in the PolyHIPE scaffolds fabricated with different W/O ratio.

The mercury intrusion porosimetry results in Figure 3 showed that the total pore volume of the polyHIPE materials increased from 7.02 to 9.61 mL/g while the W/O increased from 80/20 to 90/10. The pore size of the interconnected “windows” sharply increased from 0.31 μm to 2.61 μm while the percentage of inner aqueous phase increased from 80% to 90%, as shown in Table 2. From the mercury intrusion porosimetry data, the porosity for the polyHIPE materials prepared with different W/O ratios was as high as 89.8–95.3%. The increases in the size of voids and “windows”, as well as the pore volume and porosity were likely due to the fact that large droplets could be formed at a higher W/O ratio. As the water fraction increases, the surfactant must stabilize an increasingly large water-oil interfacial area. With a decrease the water-oil interfacial area, the average water droplet size would greatly increase, which resulted in an increase in the void size of the resultant polyHIPE materials with a higher W/O ratio. Meanwhile, the skeletal framework (continuous phase in HIPE) of the solid poly(St-DVB) became progressively thinner, which resulted from the concomitant thinning of the continuous phase film around the aqueous droplets. Thus, the increase of the water fraction resulted in an increase in the size of “windows”.

**Figure 4:**
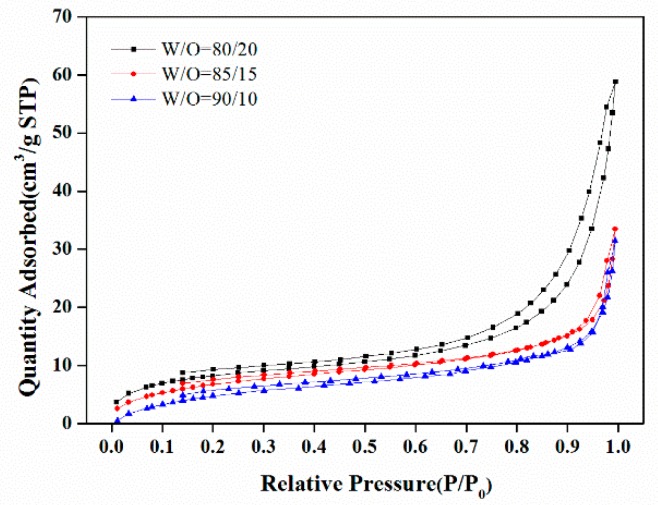
N_2_ adsorption-desorption curves of the polyHIPE scaffolds fabricated with different W/O ratio.

Figure 4 showed the nitrogen adsorption-desorption curves of the polyHIPE materials with varied W/O ratio. The BET surface areas, as cumulated with the nitrogen adsorption isotherms, were 30.5 m^2^/g, 26.5 m^2^/g and 24.6 m^2^/g for the polyHIPE materials prepared with W/O ratio of 80/20, 85/15 and 90/10, respectively. A slight decrease in the BET surface area was observed with the W/O ratio increased. The average pore sizes of <Dw>, which were obtained from BJH treatment the of nitrogen adsorption data, were 15.2 nm, 19.0 nm and 21.5 nm for the polyHIPE materials with an increased W/O ratio, respectively. The changes of BET surface area and <Dw> likely resulted from the phase separation in the polymerization process. At a higher W/O ratio, the emulsifier is less able to effectively prevent the water from entering the continuous phase during polymerization, which resulted in the premature phase separation during the polymerization process and therefore a larger <Dw> and a lower BET surface area.

#### 3.1.2. The Emulsifier Amount

**Figure 5:**
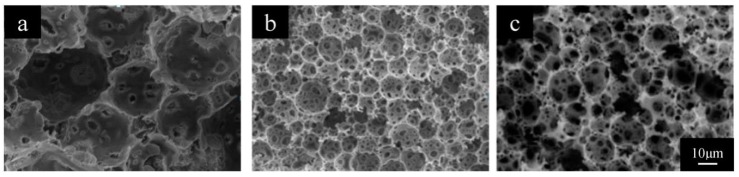
SEM images of PolyHIPE Scaffolds fabricated with emulsifier amount of (**a**) 30%, (**b**) 40% and (**c**) 50%.

Figure 5 showed the SEM images of polyHIPE materials prepared with different amount of emulsifier. With a higher ratio of emulsifier to the monomer (e.g., E/M 40% or 50%), the polyHIPE materials exhibited open cellular porous structures with many large open voids and interconnected “windows”. However, a lot of the voids and windows turn into closed while using a lower amount of emulsifier (e.g., E/M 30%). The average void sizes were 23.7 μm, 15.8 μm and 12.0 μm for the polyHIPE materials prepared with emulsifier amounts of E/M 30%, 40% and 50%, respectively, which are listed in Table 2 (Formulations 3–5).

**Figure 6:**
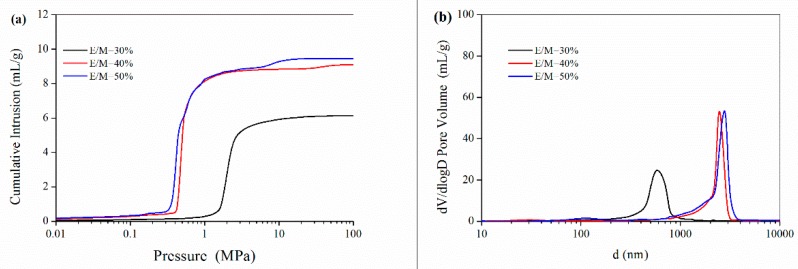
(**a**) Cumulative mercury intrusion traces and (**b**) Size distribution of the interconnected windows in the PolyHIPE scaffolds fabricated with different emulsifier amount.

The mercury intrusion porosimetry results in Figure 6a show that the pore volume increased from 6.15 mL/g to 9.61 mL/g while the emulsifier amount increasing. As seen from the pore size distribution results in Figure 6b, the average pore size of interconnected “windows” for the polyHIPE materials with 30%, 40% and 50% emulsifiers were 0.60 μm, 2.56 μm and 2.61 μm, respectively, as listed in Table 2. From the mercury intrusion porosimetry data, the porosity for the polyHIPE materials prepared with different emulsifier amount was as high as 87.9–95.3%. Notably, the high porosity and large voids would benefit these polyHIPE materials acting as scaffolds for 3D cell culture.

With an increase in the amount of Span 80 in the HIPE, the emulsifier is able to stabilize an increasingly large interfacial area. Thus, the average water droplet size became correspondingly smaller and the size of void decreased. At the same time, the skeletal framework (continuous phase in HIPE) of the solid poly(St-DVB) becomes progressively thinner, which causes the concomitant thinning of the continuous phase film around the aqueous droplets, which would result in an increase in the size of the window with a higher emulsifier amount.

**Figure 7:**
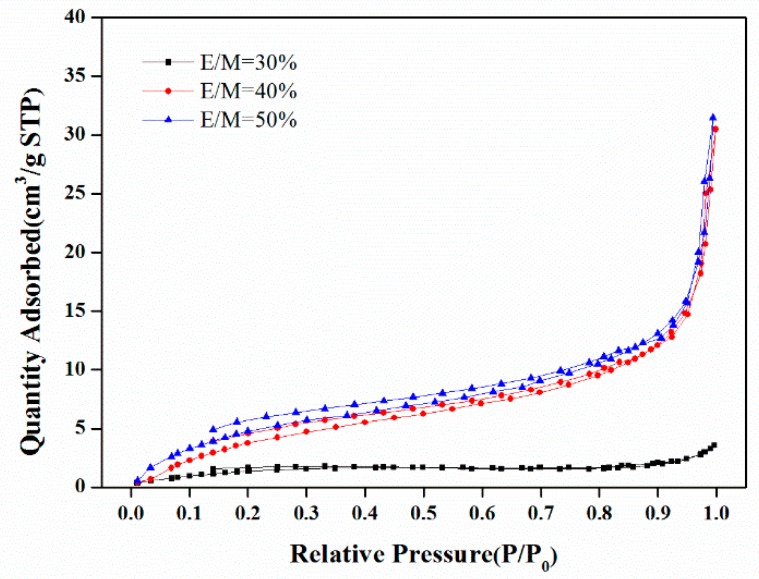
N_2_ adsorption-desorption curves of the polyHIPE scaffolds fabricated with different emulsifier amount.

The nitrogen adsorption-desorption curves of the polyHIPE scaffolds were shown in Figure 7. With the increase of emulsifier amount from E/M 30% to E/M 50%, the BET surface areas increased largely from 6.3 m^2^/g to 24.6 m^2^/g, and the <Dw> decreased from 58.6 nm to 21.5 nm, as determined from the nitrogen adsorption-desorption data, which are listed in Table 2. When more Span80 is used, more surfactant molecules are located at the oil–water interface, which leads to the formation of denser and more compact interfacial films. These films can retard the passage of the water phase (the dispersed phase) through the oil phase (the continuous phase) and prevent the premature phase separation of the growing polymeric network, resulting in a larger surface area and a decrease in the pore size of <Dw>.

### 3.2. Cell Proliferation on the PolyHIPE Scaffolds

Cell proliferation is an important prerequisite to evaluate the cytotoxicity of cigarette smoke. Herein, the polyHIPE material with a high porosity and large voids connected with windows was used as a scaffold for 3D cell proliferation. A549 cells were cultured on the polyHIPE scaffolds and determined the 3D cell proliferation ability using the CKK-8 assay.

**Figure 8:**
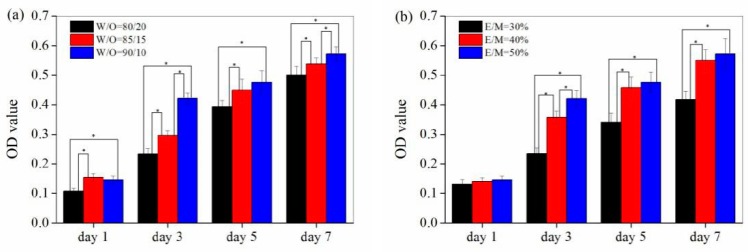
Cell Proliferation curves of A549 cells on different scaffolds. (**a**) PolyHIPE scaffolds fabricated with different W/O ratio. (**b**) PolyHIPE scaffolds fabricated with different emulsifier amount. * Significantly difference *p* < 0.05 (equal variance *t*-test).

As shown in Figure 8, a significant increase in optical density (OD) value was observed with the increase of culturing time, indicating that cells proliferated steadily on the surface and/or inside the polyHIPE scaffolds with the increase of culturing time. Furthermore, a larger optical density (OD) value was observed for the polyHIPE scaffold prepared with a higher W/O ratio or a larger emulsifier amount (E/M ratio), indicating that a promoted cell proliferation could be achieved for the polyHIPE scaffolds with a higher W/O ratio and emulsifier amount. These results were likely to be associated with the difference in the porous structure of the polyHIPE scaffolds. Herein, polyHIPE scaffolds served as ECMs for cell adhesion, proliferation and apoptosis, helping the cells to maintain a more representative structure, gene expression and function. The sizes of the void and window are essential for the cell proliferation since they could affect the nutrient and waste transfer for cell growing. Larger void and connected windows, as well as the high porosity, would benefit the nutrient and waste transfer and therefore resulted in a promoted cell proliferation. Therefore, the polyHIPE scaffold prepared with a higher W/O ratio and emulsifier amount, which has a higher porosity and larger void and connected window size, could greatly promote the cell proliferation.

### 3.3. Application of the PolyHIPE Scaffolds in Cytotoxicity Evaluation of Cigarette Smoke

Due to the high porosity, large void and connected window size, and a great promotion of cell proliferation, the polyHIPE materials prepared with a W/O ratio of 90/10 and an emulsifier amount of E/M 50% was used to construct a 3D cell cytotoxicity evaluation system, in which the cigarette smoke cytotoxicity was evaluated. For comparison, the cytotoxicity evaluations of cigarette smoke were also performed with routine 2D cell culture directly on the tissue culture plate (2D-TCP).

**Figure 9:**
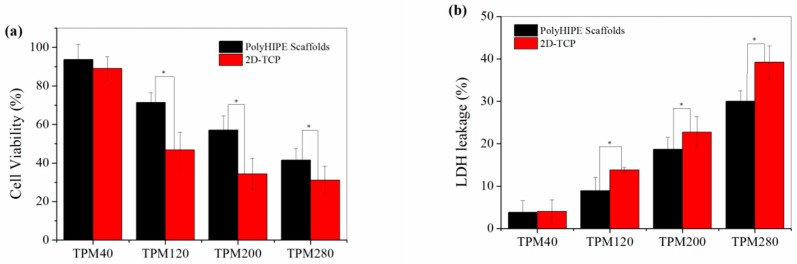
Cytotoxicity evaluation of cigarette smoke of 3R4F using polyHIPE scaffolds for 3D cell culture and the 2D cell culture directly on tissue culture plate (2D-TCP). (**a**) Cell viability determined by CKK-8 assay under TPM expose with 40–280 μg/mL dose; (**b**) LDH leakage under TPM expose with 40–280 μg/mL dose. * Significantly difference *p* < 0.05 (equal variance *t*-test).

Figure 9a shows the cell viability as determined by the CKK-8 assay under the smoke TPM exposure with an increased exposure dose. Using the polyHIPE scaffold-based 3D cell cytotoxicity evaluation system, the cell viability of A549 decreased after smoke TPM treatment of as low as 40 μg/mL TPM exposure. Cell viability decreased to 71.4%, 57.1% and 41.5% after 24 h exposure to 120, 200, 280 μg/mL TPM, respectively, showing a dose-dependent cytotoxicity of smoke TPM exposure. However, much lower cell viabilities were observed when using the traditional 2-D cell cytotoxicity evaluation system. For example, cell viability in the 2D platform decreased to 46.8%, 34.3% and 31.1% after 24 h exposure to 120, 200, 280 μg/mL TPM, which is much lower than that in the polyHIPE-based 3D platform, indicating that the cytotoxicity of smoke TPM against A549 is lower in the 3D cell platform than the traditional 2D system. Lactate dehydrogenase (LDH) release from cells under the smoke TPM exposure was also analyzed and the results were shown in Figure 9b. The LDH released 3.8–30.0% under the expose of smoke TPM with a dose of 40–280 μg/mL in the 3D cell cytotoxicity evaluation platform with polyHIPE scaffolds. However, a larger LDH leakage of 4.0–39.2% was observed in the 2D platform with the same exposure dose of smoke TPM. These results also indicated that the smoke cytotoxicity against A549 is lower in 3D cell platform than that of the 2D system. These results are consistent with the reported cytotoxicity evaluation results of cigarette smoke based on a self-assembling peptide nanofiber 3D cell culture system [40,41]. The lower cytotoxicity of smoke TPM in the 3D cell system is probably due to that cells could form a 3D gradient similar to in vivo in the 3D platform, which would not only affect the transfer of nutrients and wastes, but also the permeability of TPM, benefiting a higher cell viability.

Considering the controlled pore structure and the enhanced simulation of complex tissue architectures, the polystyrene-based polyHIPE scaffolds would be a potential candidate for 3D cell culture and application in the cytotoxicity evaluation of cigarette smoke.

## 4. Conclusions

Polystyrene-based polyHIPE materials were prepared by the HIPE templating method. The resultant polyHIPE materials were found to have an open-cellular morphology and high porosity. The pore structure of the polystyrene-based polyHIPE could be well adjusted by varying the W/O ratio and the emulsifier amount. The 3D cell culture and cell proliferation results showed that the open-cellular structure of the polyHIPE materials benefited the cells proliferated on the 3D scaffolds. The polyHIPE scaffold prepared with a higher W/O ratio of 90/10 and an emulsifier amount of E/M 50% exhibited a higher porosity, larger void and connected window size, greatly promoting cell proliferation. Finally, a 3D cell cytotoxicity evaluation system was constructed with the polystyrene-based polyHIPE materials as scaffolds, in which the cigarette smoke cytotoxicity was evaluated and compared with the traditional 2D system. The results showed that the smoke cytotoxicity against A549 is much lower in the 3D cell platform compared to the traditional 2D system. The study demonstrated the great potential of the polyHIPE scaffolds for 3D cell culture and cytotoxicity evaluation of cigarette smoke.

## Figures and Tables

**Table 1:** Preparation parameters of the polyHIPE Scaffolds.

Formulation No.	St (mL)	DVB (mL)	Span80 (mL)	AIBN (g)	H_2_O (mL)	W/O ^a^	E/M ^b^
1	5.00	0.80	2.90	0.25	34.8	80/20	50%
2	5.00	0.80	2.90	0.25	49.3	85/15	50%
3	5.00	0.80	2.90	0.25	78.3	90/10	50%
4	5.00	0.80	2.30	0.25	72.9	90/10	40%
5	5.00	0.80	1.70	0.25	68.0	90/10	30%

^a^ W/O, the volume ratio of water phase to the oil phase. ^b^ E/M, the volume ratio of emulsifier (Span 80) to the monomers.

**Table 2:** Characterization of the polyHIPE materials.

Formulation No.	BET Surface Area (m^2^/g)	Pore Volume (mL) ^a^	Porosity (%) ^a^	D (μm) ^b^	D (μm) ^c^	Dw (nm) ^d^
1	30.5	7.02	89.8	6.5	0.31	15.2
2	26.5	8.49	94.8	11.2	2.00	19.0
3	24.6	9.61	95.3	12.0	2.61	21.5
4	23.7	9.08	86.4	15.8	2.56	23.2
5	6.3	6.15	87.9	23.7	0.60	58.6

^a^ Pore volume and porosity determined by Hg mercury intrusion porosimetry; ^b^ Average void diameter determined by SEM; ^c^ Average interconnected window diameter determined by Hg mercury intrusion porosimetry; ^d^ Pore size (Dw) obtained from the BJH treatment of N_2_ adsorption data.
